# Elaboration over a Discourse Facilitates Retrieval in Sentence Processing

**DOI:** 10.3389/fpsyg.2016.00374

**Published:** 2016-03-15

**Authors:** Melissa Troyer, Philip Hofmeister, Marta Kutas

**Affiliations:** ^1^Department of Cognitive Science, University of California at San DiegoLa Jolla, CA, USA; ^2^Department of Cognitive, Linguistic, and Psychological Sciences, Brown UniversityProvidence, RI, USA; ^3^Department of Neurosciences, University of California at San DiegoLa Jolla, CA, USA

**Keywords:** sentence processing, retrieval, elaboration, representational complexity, semantic memory, self-paced reading

## Abstract

Language comprehension requires access to stored knowledge and the ability to combine knowledge in new, meaningful ways. Previous work has shown that processing linguistically more complex expressions (‘*Texas cattle rancher*’ vs. ‘*rancher*’) leads to slow-downs in reading during initial processing, possibly reflecting effort in combining information. Conversely, when this information must subsequently be retrieved (as in filler-gap constructions), processing is *facilitated* for more complex expressions, possibly because more semantic cues are available during retrieval. To follow up on this hypothesis, we tested whether information distributed across a short discourse can similarly provide effective cues for retrieval. Participants read texts introducing two referents (e.g., two senators), one of whom was described in greater detail than the other (e.g., ‘*The Democrat had voted for one of the senators, and the Republican had voted for the other, a man from Ohio who was running for president*’). The final sentence (e.g., ‘*The senator who the* {*Republican*/*Democrat*}*had voted for…*’) contained a relative clause picking out either the Many-Cue referent (with ‘*Republican*’) or the One-Cue referent (with ‘*Democrat*’). We predicted facilitated retrieval (faster reading times) for the Many-Cue condition at the verb region (‘*had voted for*’), where readers could understand that ‘*The senator*’ is the object of the verb. As predicted, this pattern was observed at the retrieval region and continued throughout the rest of the sentence. Participants also completed the Author/Magazine Recognition Tests (ART/MRT; Stanovich and West, 1989), providing a proxy for world knowledge. Since higher ART/MRT scores may index (a) greater experience accessing relevant knowledge and/or (b) richer/more highly structured representations in semantic memory, we predicted it would be positively associated with effects of elaboration on retrieval. We did not observe the predicted interaction between ART/MRT scores and Cue condition at the retrieval region, though ART/MRT interacted with Cue condition in other locations in the sentence. In sum, we found that providing more elaborative information over the course of a text can facilitate retrieval for referents, consistent with a framework in which referential elaboration over a discourse and not just local linguistic information directly impacts information retrieval during sentence processing.

## Introduction

Real-world knowledge is activated rapidly and richly in language comprehension (e.g., Kutas and Federmeier, 2000; DeLong et al., 2005; Metusalem et al., 2012). Knowledge about events, actions, and entities in the world can rapidly affect people’s expectations about upcoming linguistic information (e.g., Kamide et al., 2003; DeLong et al., 2005; Borovsky et al., 2012). What’s more, real-world knowledge use during language comprehension is dynamic, and new information can update, amend, or contradict prior information.

The ability to access this continually updated information depends on a number of factors, including the linguistic context. For instance, Bransford and Johnson (1972) provided participants with labeled and unlabeled versions of prose passages. One passage described an activity in which people typically arrange things into groups, go to the appropriate facilities, and perform a routine where a mistake may be rather expensive. Participants who initially received a label (e.g., *washing clothes*) had better memory for the passages. Similar effects have been observed when people are asked to remember information that has been causally linked [e.g., (1) someone needing change because (2) they need to do their laundry] compared to unrelated information (Smith et al., 1978; see also Bradshaw and Anderson, 1982). These findings, among others, demonstrate how language comprehension is fundamentally linked to the supporting knowledge structures, or schema, that are available to the comprehender (Radvansky and Zacks, 1991).

In addition to affecting oﬄine processes like explicit memory, the availability of related linguistic information in a sentence (e.g., the number of adjectives modifying a noun) appears to affect online sentence processing (Hofmeister, 2011; Hofmeister and Vasishth, 2014). Modifying a referent’s description with a likely attribute description (e.g., *a ruthless dictator*) leads to faster reading times at words that trigger retrieval of this discourse referent, compared to a referring expression with no modifiers. However, modification with attributes that are unlikely based on real-world knowledge (e.g., *a lovable dictator*) does not lead to the same facilitation, compared to the baseline condition (Hofmeister, 2011). In short, re-accessing previously encoded content appears to be influenced by the ability to access and use prior world knowledge in both online and oﬄine language tasks.

Here, we test whether providing more (vs. less) information about referents across a discourse similarly can increase the ease of language comprehension when these referents are subsequently referred to. In previous work on the role of elaboration in sentence processing (Hofmeister, 2011; Hofmeister and Vasishth, 2014), the syntactic constructions used to investigate elaboration and retrieval were limited to pre-nominal modification and filler-gap dependencies that linked elements within a sentence. A natural question is whether the effects observed in such environments are specific to that particular combination of encoding and retrieval conditions, or whether elaboration can facilitate online language comprehension more generally. This work therefore examines the generality of conceptual elaboration effects in language processing.

Given variability in knowledge due to individual experience, it is likely that individuals also differ from one another in their ability to access and use any particular knowledge structure. If the performance profiles described above depend significantly on the availability of existing knowledge structures, then individual profiles ought to vary as a function of their experience accessing relevant knowledge or the availability of richer or highly structured representations in memory. Before outlining the current experiment, we briefly describe work underscoring the importance of world knowledge for guiding online language comprehension.

When understanding sentences, people seem to anticipate upcoming information based on the relationship between current linguistic information and prior world knowledge (e.g., Tanenhaus et al., 1995; Kamide et al., 2003; Borovsky et al., 2012). For instance, if a listener hears ‘*The pirate chases the…*,’ it is reasonable for her to expect that the sentence will continue with something that a pirate (the agent) might chase (the action verb), such as a ship. Visual world eye-tracking paradigms, in which participants listen to spoken language while looking at images of items on a computer screen, have shown that both children and adults are sensitive to this type of information and use it to anticipate upcoming linguistic content (e.g., Kamide et al., 2003; Borovsky et al., 2012, 2013; Troyer and Borovsky, 2015).

In addition to eye-tracking paradigms, event-related brain potential (ERP) experiments support the role of real-world knowledge in guiding language comprehension. For instance, the N400 ERP component, whose amplitude is modulated by the semantic fit of meaningful input with prior context (Kutas and Hillyard, 1980, 1984; Kutas and Federmeier, 2000; see Kutas and Federmeier, 2011, for a recent review), is sensitive not only to fit of (or expectations about) semantic information but also to incoming information as it relates to individuals’ real-world knowledge (Hagoort et al., 2004; Nieuwland and Van Berkum, 2006; Hald et al., 2007; Filik and Leuthold, 2013). For instance, Hagoort et al. (2004) presented participants with sentences drawing upon world knowledge, such as the fact that the color of Dutch trains is yellow. They found reduced N400 amplitude to words like ‘*yellow*’ in the sentence ‘*Dutch trains are yellow and very crowded*’ compared to sentences like ‘*Dutch trains are sour and very crowded*’ (where ‘*sour*’ is semantically inconsistent) and ‘*Dutch trains are white and very crowded*’ (where ‘*white*’ is semantically consistent but inconsistent with world knowledge about Dutch trains). These findings support the notion that experienced-based world knowledge (Dutch trains are yellow) affects language comprehension with the same time course as (and possibly via similar mechanisms to) semantic information (trains cannot be sour).

Furthermore, Metusalem et al. (2012) showed that rich information about events in the world is available during language comprehension. In their study, people read short scenarios about events—for example, a football game: ‘*Jeremy is a great athlete despite being prone to injury. During his last high school football game, he was knocked unconscious twice. That still didn’t keep him from scoring the winning {TOUCHDOWN/HELMET/LICENSE} with only seconds remaining.*’ Unsurprisingly, N400 amplitude was reduced to predictable words fitting both with event-related information and with the semantics of the sentence (like ‘*touchdown*’) compared to anomalous words (like ‘*license*’). Critically, N400 amplitude was intermediate to words which were not plausible continuations of the sentence but which were event-related (e.g., ‘*helmet*,’ which is situationally related to football). These findings suggest that a rich landscape of knowledge can be rapidly activated during language comprehension, likely contributing to the flexibility of language comprehension.

Participants in the Metusalem et al. (2012) study also completed two tasks called the Author and Magazine Recognition Tests (ART and MRT, respectively), which require participants to select the authors and magazines that they recognize from lists containing both real and false examples (Stanovich and West, 1989). These tests provide an estimate of print experience, and the authors suggested that, by proxy, higher performance on the ART/MRT could reflect richer world knowledge. Indeed, performance on the ART/MRT predicts measures of declarative knowledge, including tests of cultural literacy recognition (*r*s = 0.53 - 0.72; West et al., 1993; Stanovich et al., 1995); tests about history and literature knowledge (*r*s = 0.59 - 0.62; Stanovich and Cunningham, 1992); a range of tests about cultural and practical knowledge (*r*s = 0.53 - 0.85, Stanovich and Cunningham, 1993); and, in children, the General Information subtest of the Peabody Individual Achievement Test (using a modified Title Recognition Test for Children; *r* = 0.43; Cunningham and Stanovich, 1991). If prior world knowledge influences access to event-related information, then N400 amplitude might vary with performance on the ART/MRT. The authors found that scoring higher on the ART and MRT was associated with a greater numerical reduction in N400 amplitude for implausible, yet event-related, continuations (e.g., ‘*helmet*,’ in the example above), compared to participants who scored lower on the ART/MRT. However, the authors were unable to draw strong conclusions about the relationship between the N400 and scores on the ART/MRT, partly due to the number of participants (*N* = 30), which is relatively low for examining individual differences.

In combination with prior world knowledge, new information—for example, information encountered in the current discourse—can be exploited rapidly to aid future language processing. For example, Nieuwland and Van Berkum (2006) presented participants with short texts in which they ascribed human-like properties (e.g., the ability to fall in love) to typically inanimate objects (e.g., peanuts). In their experiments, the N400 was sensitive to these newly learned features, suggesting that people easily updated their mental models of the discourse to include these properties.

The current work investigates how variability in the amount of recently encountered information, providing elaboration of a referent, affects subsequent access. This work extends recent findings from self-paced reading studies that suggest that longer or more semantically complex linguistic representations of referents can facilitate subsequent access to those referents (Hofmeister, 2011; Hofmeister and Vasishth, 2014). For instance, Hofmeister (2011) asked participants to read (word-by-word) sentences in which a critical noun was described by zero, one, or two adjectives (low, mid, and high complexity conditions, respectively). Participants might read, ‘*It was a [famous (deaf)]* ***sculptor*** *that the aristocrats at the gallery ridiculed during the exclusive art show*.’ At a subsequent critical verb (e.g., ‘*ridiculed*’), the critical noun had to be understood as the grammatical object of the verb. In order to access this information, participants must somehow retrieve information about the initial noun (e.g., ‘*sculptor*’). Hofmeister (2011) reported decreased reading times during (or in some cases, immediately following) the critical verb for items in the highest-complexity condition (i.e., where critical nouns were preceded by two adjectives) compared to the other conditions. In similar experiments, such findings also were observed for nouns which were semantically richer/more specific (e.g., ‘*soldier*’) compared to less rich/less specific (e.g., ‘*person*’). Hofmeister (2011) interpreted these results as showing that additional semantic (and possibly syntactic) features of a linguistic representation led to facilitated retrieval of the information later in the sentence.

Studies like those of Hofmeister (2011) and Hofmeister and Vasishth (2014) have primarily focused on pre-nominal descriptors (‘*Texas cattle rancher*’) or differences in the semantic specificity/richness of a single word (‘*soldier*’ vs. ‘*person*’) but have not explored the roles of other types of descriptions across a discourse. Pre-nominal adjectives are likely to change the processing of an upcoming noun for multiple reasons. First, in an information-theoretic sense, pre-nominal modification can lower the entropy of (or uncertainty about) the upcoming noun. Second, modifiers might be predictive of the noun for other reasons such as semantic relatedness (consider the relationship between the three words ‘*Texas*,’ ‘*cattle*,’ and ‘*rancher*,’ for example). And finally, pre-nominal modification entails a specific type of syntactic relationship between modifiers and the noun, with the entire bundle of linguistic information [modifier(s) + noun] constituting a phrasal unit.

In the current study, we investigate how complex descriptions impact the subsequent retrieval of information about referents in language comprehension across sentence boundaries. We vary the additional linguistic information not in adjectival modifiers directly preceding the noun, but using post-nominal modification across multiple sentences in a short discourse. We predicted that providing higher-complexity descriptions about referents would make it easier for participants to process subsequent language referring to those referents compared to referents with linguistically simpler descriptions. Such a finding would indicate that conceptual complexity, above and beyond the phrasal unit, can influence retrieval in real-time language comprehension.

We also asked participants to complete a simple test designed to assess print exposure, which has been used as a proxy for real-world knowledge (e.g., Metusalem et al., 2012). We predicted that participants with greater world knowledge would be able to more effectively make use of additional information—possibly due to richer networks of conceptual representations and/or more effective access to relevant conceptual information. We therefore predicted these participants would be more likely to show effects of linguistic complexity at subsequent retrieval sites.

## Materials and Methods

### Participants

A total of 101 participants, ages 18–29 (*M* = 20.7, 77 women) took part in the experiment. Participants were excluded from analysis if their overall accuracy on comprehension questions was less than 70%. This resulted in the exclusion of nine participants, for a total of 92 participants in the final dataset. Participants were students at UCSD who reported that they were native English speakers. They received partial class credit for participation. All participants provided informed consent for the study, which was approved by the University of California, San Diego Institutional Review Board.

### Design and Materials

The materials for the study were 24 experimental items and 36 filler items of similar length and syntactic complexity. The majority of our materials were created by modifying materials from Fedorenko et al. (2012). A full listing of the experimental and filler items can be found in the Appendix in the Supplementary Data Sheet. Each item consisted of a short text of three sentences. All items began with two sentences, which were presented and read (self-paced) as whole sentences. The third sentence was presented word-by-word, using a moving-window self-paced reading paradigm (Just et al., 1982). Filler items were constructed to be similar to experimental items in length and content.

For experimental items, the first sentence always introduced four individuals, two of whom were referred to using the same noun (e.g., ‘*senator*,’ in the example below). The second sentence always described relationships between the first two individuals (e.g., the two senators) and the second two (e.g., the Democrat and the Republican), with one of the first two individuals being described in more detail more than the other. In the third and final sentence, the second noun was varied to unambiguously pick out a referent for its object. In the example below, for instance, ‘*The senator who the Republican had voted for*’ would refer to the senator from Ohio who was running for president (the Many-Cue condition), while ‘*The senator who the Democrat had voted for*’ would refer to the other senator (the One-Cue condition).

(1) Sentence 1: Two senators were arguing with a Democrat and a Republican after a big debate.
Sentence 2: The Democrat had voted for one of the senators, and the Republican had voted for the other, a man from Ohio who was running for president.
Sentence 3: The senator who the {Republican/Democrat} had voted for was picking a fight about health care reform.

As described above, Cue condition refers to the presence or absence of additional descriptive information in the second sentence. To mitigate any effect of recency of information on reading times, we also created a second version of the materials in which the Many-Cue item came earlier than the One-Cue item. For example, in the second version of the example shown in (1), the second sentence would read, ‘*The Democrat had voted for one of the senators, a man from Ohio who was running for president, and the Republican had voted for the other*.’ The factor Mention Order refers to whether the critical item (i.e., the object of the relative clause in Sentence 3) was mentioned relatively early or relatively late in the second sentence. In the example above (1), the information is Early for the One-Cue condition (i.e., ‘*The Democrat had voted for one of the senators*’) but Late for the Many-Cue condition (i.e., ‘*The Republican had voted for one of the senators*’). The design was therefore a 2 × 2: Cue condition (Many-Cue, One-Cue) and Mention Order (Early, Late). This resulted in four lists, randomized across participants according to a Latin-square design such that no participant saw the same exact order of experimental and filler items.

Finally, each text was followed by a comprehension question, which participants answered with *yes* or *no* by key press. Across the experiment, comprehension questions queried each of the three sentences in a text so that a third focused on Sentence 1, a third on Sentence 2, and a third on Sentence 3. Half of the sentences were answered correctly with *no* and half with *yes*. For the example above in (1), the comprehension question asked about the first sentence and was correctly answered with *yes*: *Were the senators arguing before a big debate?* Similarly, filler questions asked about either the first, second, or third sentence, in equal proportions. Half of each set were correctly answered with yes, and half with no.

### Author and Magazine Recognition Tests

Prior to testing, participants also completed an updated version of the ART and the MRT (Stanovich and West, 1989). These tasks were designed to provide a simple yet powerful way to estimate print experience and, by proxy, world knowledge. Previous work has found correlations in the range of r = 0.5 – 0.8 between ART/MRT and many measures of declarative/cultural knowledge (Cunningham and Stanovich, 1991; Stanovich and Cunningham, 1992, 1993; West et al., 1993; Stanovich et al., 1995); in addition, both tests correlate (rs = 0.3 – 0.4) with measures of reading comprehension, and the ART also correlates with measures of orthographic and phonological processing (Stanovich and West, 1989). Participants were given a printed list of 80 potential author names (ART) and 80 potential magazine titles (MRT; presented separately) and were asked to put a check mark next to the ones they knew to be true authors/magazines. In actuality, only half were real authors/magazines. Participants were asked to avoid guessing because some of the names on the lists were not actual authors/magazines. Scores for these tasks were calculated by summing the number of hits (correct items checked) minus the number of false alarms (checked items which were incorrect). The scores for both tasks were computed separately but combined (summed) for analyses.

### Procedure

We used Linger (version 2.88) by Doug Rohde to collect self-paced reading data. For this part of the experiment, participants were instructed that they would be reading short texts made up of three sentences and that they should read the sentences for content, as there would be comprehension questions following each text. They were provided with examples and familiarized with the task before they began, including practice on two items very similar to those used in the study, preceded by a few simpler examples of word-by-word self-paced reading.

Accuracy was computed on the fly and in aggregate in subsequent analyses. If participants responded incorrectly, a warning flashed on the screen to encourage them to try harder to answer correctly on subsequent questions. Participants were given a break halfway through the experiment and instructed to take short breaks as needed in between items.

Following testing, participants completed an exit questionnaire including questions about the ease of the experiment. The experiment was typically completed in under an hour.

### Analysis

Although the final sentence of each text was presented word by word, five regions were created, the last four of which were analyzed (an example is demarcated below). Region 1 always consisted of a noun phrase (two words); Region 2 was the start of the relative clause (three words); Region 3 was the verb phrase of the relative clause (1–3 words); Region 4 was the matrix verb phrase region (2–5 words); and Region 5 was a final region including direct objects, adverbials, or prepositional phrases (2–7 words).

(2) The senator/who the Republican/had voted for/was picking a fight/about health care reform.

For the primary analyses, we first identified any trial containing single-word responses that were less than 100 ms or greater than 5000 ms and removed these trials from subsequent analysis, affecting less than 1% of the data. Next, for each trial, RTs for words within a region were averaged. These averaged RTs were then log-transformed, and data points falling more or less than 2.5 SDs from the mean (by region and condition) were eliminated, affecting ~2.5% of the data.

Statistical analyses used linear mixed-effects models (Baayen, 2008) incorporating random effects for both items and subjects as well as fixed effects of Cue condition, Mention Order, and Spillover (log RT of the preceding region) as fixed effects, unless otherwise indicated. In addition, we included by-subjects and by-items random slopes for Cue condition, as this was our primary independent variable of interest. All analyses were performed in the statistical programming environment R.

## Results

### Self-Paced Reading

**FIGURE 1:**
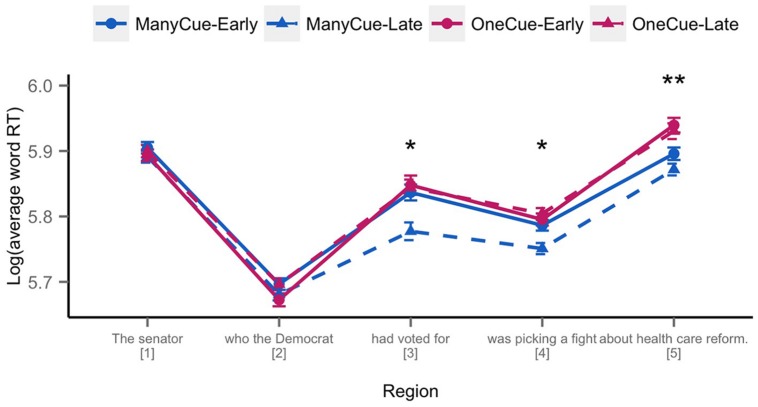
**Log average word reading times by region for sentence 3.** Errors bars represent by-subject standard errors of the mean. There was a main effect of Cue condition at Regions 3–5 (Many-Cue > One-Cue; ^*^p < 0.05; **p < 0.001). See **Table 1** for full model statistics.

Mean log reading times by region are shown in **Figure 1**, and full model estimates and statistics are provided in **Table 1**.

**Table 1:** Full model estimates and statistics for reading times from the final sentence.

Region	Effect	Estimate	Standard Error	*t*-value	*p*-value
Region 2	**(Intercept)**	**5.693**	**0.023**	**247.15**	**0.000**
	Cue condition	0.000	0.007	-0.059	0.953
	Mention Order	-0.003	0.005	-0.656	0.512
	**Cue × Order**	-**0.011**	**0.005**	-**2.055**	**0.040**
Region 3	**(Intercept)**	**5.834**	**0.026**	**222.01**	**0.000**
	**Cue condition**	**0.019**	**0.008**	**2.394**	**0.025**
	Mention Order	0.014	0.008	1.794	0.073
	Cue × Order	-0.013	0.008	-1.680	0.093
Region 4	**(Intercept)**	**5.786**	**0.022**	**264.604**	**0.000**
	**Cue condition**	**0.016**	**0.006**	**2.632**	**0.015**
	Mention Order	0.005	0.005	0.899	0.369
	Cue × Order	-0.011	0.005	-1.953	0.051
Region 5	**(Intercept)**	**5.916**	**0.025**	**238.968**	**0.000**
	**Cue condition**	**0.026**	**0.006**	**4.074**	**0.000**
	Mention Order	0.008	0.005	1.639	0.101
	Cue × Order	-0.003	0.005	-0.544	0.586

*Statistically significant predictors (*p* < 0.05) are in bold.*

At the second region (which is the point at which the noun phrase ‘*The senator*’ begins to be disambiguated), we observed no main effect of Cue condition or Mention Order, but there was a significant interaction of the two (β = -0.011, *SE* = 0.005, *t* = -2.055, *p* < 0.05). Visual inspection revealed this interaction appeared to be driven by slower reading times for conditions from Version 1 (Many-Late, One-Early) compared to Version 2 (Many-Early, One-Late; see above for an example of Version 1 vs. Version 2 of the materials). A follow-up analysis with Version (V1, V2) as fixed effects and Subject and Item as random effects indicated this was the case, with a significant difference between the two (β = -0.011, *SE* = 0.005, *t* = -2.04, *p* < 0.05).

Region 3 was the retrieval region where we predicted a main effect of Cue condition. Here, we observed the predicted main effect of Cue condition, with faster reading times in the Many-Cue compared to the One-Cue condition (β = 0.019, *SE* = 0.008, *t* = 2.394, *p* < 0.05). In addition, we also observed a marginal effect of Mention Order, with relatively Late information leading to faster reading times compared to Early information (*p* = 0.07) as well as a marginal interaction of Cue and Mention Order (*p* = 0.09).

The effect of Cue condition persisted into both Regions 4 (β = 0.016, *SE* = 0.006, *t* = 2.632, *p* < 0.05) and 5 (β = 0.026, *SE* = 0.006, *t* = 4.074, *p* < 0.001). No significant main effects or interactions with Mention Order were observed in either region, though there was a marginal interaction between Cue and Order in Region 4 (*p* = 0.05).

### ART/MRT Scores

Scores on the ART and MRT were calculated separately and then summed to create a single composite score. For the ART, scores ranged from -5 (one participant checked more incorrect items than correct items, leading to the negative score) to 25, with a mean of 7.28 (*SD* = 3.87). Scores for the MRT ranged from 1 to 20, with a mean of 7.97 (*SD* = 3.83). The two tasks were positively correlated (*r* = 0.415, *p* < 0.0001). When combined by summation, the mean composite score was 15.25 (*SD* = 6.47).

### Comprehension Question Accuracies

Comprehension questions were included primarily to encourage participants to read the texts carefully. Comprehension question accuracy was 88.32% (*SD* = 6.14%) for filler materials. Analyses using mixed-effects logistic regression (with Cue condition and Mention Order as fixed effects and Subject and Item as random effects) revealed that accuracy did not differ as a function of Cue condition or Mention Order, with a mean of 79.35% (*SD* = 14.80%) for the Many-Cue condition and a mean of 77.26% (*SD* = 13.82%) for the One-Cue condition. We therefore observed that our manipulation of interest, Cue condition, had no measurable effect on oﬄine comprehension accuracies.

Accuracies were also analyzed by the type of question, that is, whether the question asked about the first, second, or third sentence. Mixed-effects logistic regression with question type (first, second, third sentence) as a fixed effect and Subjects and Items as random effects revealed that questions about the second sentence (*M* = 70.92%, *SD* = 20.89%) were answered less accurately than questions about the final sentence (*M* = 84.51%, *SD* = 13.54%; β = -0.46, *SE* = 0.17, z = -2.75, *p* < 0.01), though the difference between questions about the first sentence (*M* = 79.48%, *SD* = 14.30%) and second sentence did not reach significance (*p* = 0.14). This pattern likely reflects the fact that the second sentence was the most complex/longest of the three sentences.

### Relationship between Reading Times and ART/MRT

We predicted that individuals scoring higher on the ART/MRT, and who are therefore likely to have greater world knowledge, would show the greatest effects of Cue condition during the retrieval region. However, adding the continuous ART/MRT composite scores as a predictor did not indicate any effect of ART/MRT on reading times during Region 3 nor was there any interaction with Cue or Mention Order (all *p*s > 0.16).

However, ART/MRT scores interacted with Cue condition at an un-predicted location, in Region 2 (β = -0.002, *SE* = 0.001, *t* = -2.247, *p* < 0.05). To follow up on this interaction, we used both group comparisons based on a median split as well as a correlational analyses. Numerically, individuals scoring higher on the ART/MRT had faster reading times for the One- (*M* = 5.66 log ms, *SD* = 0.31) compared to the Many-Cue condition (*M* = 5.69 log ms, *SD* = 0.33), but individuals scoring lower on the ART/MRT had the opposite numeric pattern (One-Cue, *M* = 5.72 log ms, *SD* = 0.31; Many-Cue, *M* = 5.70, *SD* = 0.31). Mixed-effects models performed separately over each group with Cue as a fixed effect and subject and item as random effects indicated that these were only trends (*p*s = 0.09, 0.11, respectively). However, a correlational analysis of ART/MRT scores and differences between One-Cue minus Many-Cue RTs was significant, *r* = -0.216, *p* < 0.05. We had no specific predictions for any effect of Cue at this region nor any interactions with ART/MRT (but see Discussion).

In addition, ART/MRT scores interacted with Cue condition in Region 4 (β = -0.002, *SE* = 0.001, *t* = -2.172, *p* < 0.05). We again inspected both group differences and correlations between ART/MRT and reading time differences. For the higher-scoring group, there was little difference based on Cue condition (One-Cue, *M* = 5.80 log ms, *SD* = 0.33; Many-Cue, *M* = 5.79 log ms, *SD* = 0.34; difference n.s.). However, a mixed-effects model (see above) revealed a difference between the One-Cue (*M* = 5.81 log ms, *SD* = 0.32) and Many-Cue (*M* = 5.76, log ms, *SD* = 0.29) conditions for the group scoring lower on the ART/MRT (β = 0.027, *SE* = 0.008, *t* = 3.537, *p* < 0.001). The correlation between ART/MRT scores and differences between One-Cue minus Many-Cue RTs was significant (*r* = -0.283, *p* < 0.01), indicating that lower scores were associated with larger differences between conditions. Although this pattern occurred at Region 4, a region subsequent to the critical retrieval region in our experiment (Region 3), it is possible the interaction between ART/MRT and Cue condition at this region relates to continued retrieval processes. We further discuss this possibility in the discussion.

There were no other interactions with ART/MRT at any other region in this analysis.

## Discussion

### Summary of Findings

This study had two primary aims. The first was to test whether a greater amount of linguistic elaboration about a referent over a short discourse could facilitate subsequent access to that information during online language processing. If so, the second was to test whether this facilitation was greater for those with more world knowledge (determined using scores from the ART and MRT as a proxy) would lead to increased facilitation based on elaboration.

Supporting our hypothesis that elaborative information would provide more cues to retrieval, we found reduced reading times at a critical retrieval site when the referent had previously been described in more detail, albeit not more so for those with greater world knowledge. This work provides a novel contribution by suggesting that elaboration can affect retrieval-related processes in cross-sentential dependencies. These findings demonstrate the generality of elaboration effects in sentence processing (Hofmeister, 2011; Hofmeister and Vasishth, 2014).

It is particularly noteworthy that various formal syntactic theories treat anaphoric dependencies as fundamentally different than filler-gap dependencies. For instance, in transformational theories of syntax, filler-gap dependencies are licensed via cyclic movement of the filler, leaving behind a trace, whereas no such process applies to anaphoric dependencies (co-indexing provides the necessary connection; e.g., Chomsky, 1995, among many others). More importantly, the retrieval conditions in filler-gap dependencies are quite different from those in the current study. In filler-gap dependencies, the retrieval target is necessarily within the same sentence, which may limit the retrieval search space, relative to that for anaphoric dependencies. Further, the onset of a filler-gap dependency signals that the target information must be restored in the near future. That is, once a filler is encountered, a process is initiated that necessarily ends with retrieval; hence, it is predictable that the filler information will be needed again. Up to that point, the parser is actively engaged in searching for the first available integration point (Clifton and Frazier, 1989; Frazier and Clifton, 1989; Frazier and d’Arcais, 1989). This contrasts with anaphoric dependencies where there is no guarantee that a referent will ever be mentioned again—as was the case for the elaborative information presented in our short texts. In sum, anaphoric dependencies do not come with the same set of expectations or retrieval cues that accompany filler-gap dependencies. Thus, demonstrating that elaboration effects nevertheless arise in cross-sentential dependencies suggests that they are not contingent upon any of the idiosyncrasies of filler-gap dependencies.

We did not observe the predicted interaction between ART/MRT and Cue condition at Region 3. However, two unpredicted related results were the interactions between ART/MRT scores and Cue condition on reading times at Regions 2 and 4. In Region 2 (‘The senator/who the Democrat/…’), participants may begin to anticipate the upcoming object of the relative clause, though there is still ambiguity with respect to which referent will be mentioned. We tentatively speculate that differences in language experience/world knowledge (as indexed by ART/MRT scores) may affect the individual’s sensitivity to this ambiguity (or ability to predict an upcoming referent), possibly resulting in the observed interaction.

We initially hypothesized that having greater world knowledge (and higher scores on the ART/MRT, by proxy), would associate with greater ease of access for meaningful cues to retrieval. We therefore predicted greater facilitation in retrieval (at Region 3) for the Many-Cue condition, or possibly in a subsequent region, for those with greater world knowledge. However, the interaction between Cue and ART/MRT scores which we observed at Region 4 did not support our hypothesis; rather, individuals with lower ART/MRT scores drove effects of Cue condition in this region, with lower reading times associated with the Many-Cue compared to the One-Cue condition. One possibility is that for our materials, having more information benefited those with less language experience/less knowledge more, meaning that the group scoring lower on ART/MRT was able to benefit from the additional information in the Many-Cue condition while the higher-scoring group showed less of a difference between conditions. Future work using more tightly controlled stimuli (e.g., with identical numbers of words in each region, with identical syntax, etc.) might shed more light on the nature of these individual differences.

Overall, we interpret our findings as evidence that having more information about a referent is beneficial during retrieval and perhaps during subsequent comprehension, as the sentence progresses and information accumulates.

### The Role of Elaboration in Online Sentence Processing

Work by Hofmeister (2011) and Hofmeister and Vasishth (2014) has shown that under many circumstances, elaborative information, typically in the form of adjectives preceding a noun, increases processing times at the point of encoding (at the noun) but facilitates processing times at a subsequent dependency. This finding holds for words which are more elaborated in the sense that they are semantically richer (e.g., ‘*soldier*’ is richer than ‘*person*’), but it does not hold when adjectives preceding a noun are atypical descriptors (e.g., ‘*ruthless military dictator*’ is typical but ‘*lovable military dictator*’ is not). Here, we add to this literature by showing that elaborative information presented across multiple sentences, and not just locally (at the point of modifying a noun, for example), can facilitate subsequent access to or retrieval of that information.

What may account for the benefit of retrieving representations that have relatively many features associated with them, even across discourse boundaries? On one hand, such effects are surprising since it would seem to imply that more content must be retrieved. On the other, these effects align naturally with several non-mutually exclusive hypotheses about the nature of memory retrieval in language processing. For instance, in the cue-based retrieval model of Lewis and Vasishth (2005), the efficacy of retrieval for some item in memory is driven partly by its retrieval history, i.e., how many times an item has been restored and how recently. Modifying a word or phrase that has been encoded in the past reactivates that item, leading to an increase in its activation. This reactivation process can even arguably offset any effects of time-based decay, giving rise to so-called anti-locality effects (Vasishth and Lewis, 2006). From this point of view, the increased ease of retrieval observed in Regions 3–5 is ascribable to a boosted level of activation of the target either prior to retrieval, or possibly during retrieval, as relevant cues spread activation to other cues (see Hofmeister, 2011). A separate, though not mutually exclusive, view suggests that adding semantic features to a discourse referent typically gives rise to a conceptually unique representation in the current discourse context. The advantage of this elaboration is manifested at the retrieval region, as the broader memory literature demonstrates a robust memory advantage for targets with contextually unique features (Moscovitch and Craik, 1976; Fisher and Craik, 1977; Jacoby and Craik, 1979; Hunt and Worthen, 2006; Gallo et al., 2008). In essence, adding details about a person or event increases the likelihood that this entity bears conceptual features that no other memory item (or very few others) shares, reducing the chance for similarity-based interference at retrieval. Both of these views capture the observed effects in our experiment without adjudicating between them.

## Conclusion

The present findings are novel in showing that when (potentially) relevant semantic information is associated with a concept, it may directly impact its retrieval, even when the elaborative information is distributed across a discourse, and not just or at all in the local (within-sentence) linguistic context (as in Hofmeister, 2011; Hofmeister and Vasishth, 2014). Relatedly, one recent study found that when participants read longer descriptions (e.g., ‘*The actor who was frustrated and visibly upset*’ vs. ‘*The actress*’), they were more likely to refer back to them with a pronoun, a finding the authors attributed to enhanced prominence of the referent due to the elaboration (Karimi et al., 2014). When concepts are more elaborated, subsequent processing advantages may occur because (a) there are more semantic features available and/or (b) those features lead to increased activation levels of the concept. Our findings suggest that variability in the elaboration of referents may have relatively long-term consequences for their processing across the subsequent discourse.

## Author Contributions

All authors listed, have made substantial, direct and intellectual contribution to the work, and approved it for publication.

## Conflict of Interest Statement

The authors declare that the research was conducted in the absence of any commercial or financial relationships that could be construed as a potential conflict of interest.
