# Supplementary material for: Elaboration over a Discourse Facilitates Retrieval in Sentence Processing
Source: Front Psychol. 2016 Mar 15;7:374. doi: 10.3389/fpsyg.2016.00374 (PMC4791404; doi:10.3389/fpsyg.2016.00374)
Supplement: Supplementary file 1 [file Data_Sheet_1.DOCX]

**Appendix**

**A. Experimental Sentences**

Shown here is version 1, one-cue; version 1, many-cue, and version 2 (both Cue conditions) can be deduced following the schema provided in the text.

1. Two senators were arguing with a Democrat and a Republican after a big debate.

The Democrat had voted for one of the senators, and the Republican had voted for the other, a man from Ohio who was running for president.

The senator who the Democrat had voted for was picking a fight about health care reform.

2. Two musicians were talking to an interviewer and a newscaster during a radio talk show.

The interviewer had dated one of the musicians, and the newscaster had dated the other, a guitarist who also sang with a gospel group.

The musician who the interviewer had dated was answering most of the questions.

3. Two cashiers were discussing recent events with a customer and a supervisor at the grocery store.

The customer joked with one of the cashiers, and the supervisor joked with the other, a mother of two who often picked up extra shifts.

The cashier who the customer joked with was getting off work in two hours.

4. Two scientists were going over a new protocol with a technician and an intern in the lab.

The technician questioned one of the scientists, and the intern questioned the other, a UCLA graduate who was the head of the lab.

The scientist who the technician questioned was going to a conference in Philadelphia.

5. Two actors were having a conversation with a screenwriter and a director during the filming of a movie.

The screenwriter corrected one of the actors, and the director corrected the other, an Academy Award winner who often starred in period dramas.

The actor who the screenwriter corrected was planning to appear in his first Broadway role.

6. Two lawyers got along very well with a secretary and an accountant in their office in a big firm.

The secretary greeted one of the lawyers, and the accountant greeted the other, a new partner in the firm who was originally from the south.

The lawyer who the secretary greeted was running late that morning.

7. Two pediatricians often went over various medications with a cardiologist and a nurse on staff.

The cardiologist phoned one of the pediatricians, and the nurse phoned the other, an oncologist who specialized in leukemia.

The pediatrician who the cardiologist phoned was on call on Christmas Day.

8. Two neurologists worked together with a professor and a resident at the medical center on campus.

The professor gave advice to one of the neurologists, and the resident gave advice to the other, a newcomer who had gone to school in New York.

The neurologist who the professor gave advice to usually worked with epilepsy patients.

9. Two writers were quibbling about the point of a book with a critic and a publisher at a public event.

The critic annoyed one of the writers, and the publisher annoyed the other, a staff member at the New York Times who wrote Op-Ed pieces.

The writer who the critic annoyed had recently started to teach college writing.

10. Two teachers were talking about lesson plans with a student and the principal in the school office.

The student really liked one of the teachers, and the principal really liked the other, a Civil war buff who taught social studies.

The teacher who the student really liked was teaching eighth graders that year.

11. Two politicians were disagreeing with a journalist and a photographer after an interview.

The journalist criticized one of the politicians, and the photographer criticized the other, a Green Party member who supported gun control.

The politician who the journalist criticized was becoming popular with young voters.

12. Two inventors displayed new technology to a researcher and an organizer at a convention.

The researcher praised one of the inventors, and the organizer praised the other, an employee of Caltech who was interested in robotics.

The inventor who the researcher praised was writing a book on artificial intelligence.

13. Two congressmen squabbled about the national election with a governor and a moderator after the panel discussion.

The governor antagonized one of the congressmen, and the moderator antagonized the other, a Texan who lived on a ranch.

The congressman who the governor antagonized often voted in the minority of his party.

14. Two ambassadors were planning a trip with an interpreter and a reporter for later in the month.

The interpreter contacted one of the ambassadors, and the reporter contacted the other, a speaker of five languages who often worked abroad.

The ambassador who the interpreter contacted was traveling constantly for the next three months.

15. Two managers were examining the company's books with a programmer and an administrator after work.

The programmer interrogated one of the managers, and the administrator interrogated the other, a hard worker who often stayed for late hours.

The manager who the programmer interrogated was coming up for a big promotion.

16. Two historians were discussing an article with an anthropologist and a geographer in a group meeting.

The anthropologist challenged one of the historians, and the geographer challenged the other, an expert on Ancient Greece who studied papyrus.

The historian who the anthropologist challenged was going to Athens later that month.

17. Two counselors were chatting about teaching methods with a librarian and an aide at the high school.

The librarian offended one of the counselors, and the aide offended the other, an alum of the school who also worked at an after-school program.

The counselor who the librarian offended was starting to think about a change in careers.

18. Two sopranos were practicing their duet with a conductor and an accompanist before chorus rehearsal.

The conductor approached one of the sopranos, and the accompanist approached the other, a high school senior who was applying to Stanford.

The soprano who the conductor approached almost always got a solo in each performance.

19. Two engineers were chatting about a project with a mathematician and a physicist at a meeting.

The mathematician interrupted one of the engineers, and the physicist interrupted the other, a car buff who also loved to ride motorcycles.

The engineer who the mathematician interrupted was describing a new project for the company.

20. Two artists met up at a museum with a docent and a curator before a new exhibit.

The docent addressed one of the artists, and the curator addressed the other, a painter who also taught art at a local college.

The artist who the docent addressed was visiting the museum for the first time.

21. Two gymnasts were working out with a wrestler and a coach at the school gym.

The wrestler watched one of the gymnasts, and the coach watched the other, a national competitor who performed best on balance beam.

The gymnast who the wrestler watched was graduating at the top of her class.

22. Two caterers were talking about food with chef and a server before a big event.

The chef spoke to one of the caterers, and the server spoke to the other, an entrepreneur who was noted for her pastries.

The caterer who the chef spoke to was planning the menu for a wedding later in the week.

23. Two carpenters were working near each other with a bricklayer and a foreman at a construction site.

The bricklayer yelled to one of the carpenters, and the foreman yelled to the other, a specialist in restoration who had decades of experience.

The carpenter who the bricklayer yelled to was going to retire within the next year.

24. Two attorneys were talking shop with a client and a judge in the courtroom.

The client argued with one of the attorneys, and the judge argued with the other, a highly paid prosecutor who won nearly all her cases.

The attorney who the client argued with primarily handled criminal cases.

**B. Filler Sentences**

1. A model, a makeup artist, and two hairdressers discussed possible hairstyles during a photo shoot.

The model liked her hair straight; however, the makeup artist and the hairdressers wanted her hair to be curly.

The model got paid ten thousand dollars for every photo shoot but was stubborn and hard to work with.

2. A lifeguard, a swimmer and two surfers became good friends over the summer.

The lifeguard began dating the swimmer, and the surfers both envied them.

After a few weeks they broke up, and the surfers were secretly glad.

3. A higher school, his sister, and two cousins watched a movie together in a movie theater.

The high schooler threw popcorn at his sister and then one of the cousins hit the boy for misbehaving.

All four family members were kicked out of the movie theater.

4. A singer, a ballerina, and two violinists performed together during a concert.

One of the violinists accompanied the singer and then the ballerina joined in with a beautiful piece.

The singer was dating the ballerina, and they had been going out for two months.

5. A cheerleader, her father, and two injured players sat together at the basketball game.

The father disliked one of the injured players because he was dating his daughter.

In fact, the cheerleader's father disliked all of his daughter's boyfriends.

6. A tightrope walker, a clown, and two acrobats were bowing to the audience at the circus.

The clown opened the circus with an introductory act, and then the tightrope walker and the acrobats ended the show with an amazing finale.

When the tightrope walker was little, he did not want to be in the circus.

7. A chauffeur, a beauty queen and two PR reps sat in a limousine together.

The beauty queen and the chauffeur exchanged private looks while the PR reps both talked on their phones.

The beauty queen and the chauffeur were planning to elope together.

8. A sports psychologist, a snowboarder, and two instructors met after the big competition.

The sports psychologist gave a lecture and then the instructors showed a video of the snowboarder's race.

The snowboarder had won a bronze medal last year, and this year, he aspired to win the gold.

9. A cartoonist, a filmmaker and two producers expressed interest in producing a movie together.

The cartoonist and the filmmaker wanted to make a movie for young children; however, the producers wanted to make an R-rated movie.

Most of the filmmaker's movies are total failures on opening weekend.

10. A ski instructor, a teenager, and two young twins skied down the bunny slope together during a ski lesson.

The ski instructor and the teenager finished the course quickly; however, the twins fell down twice before the finish line.

It was the twins' first time skiing ever, and the last time the teenager had skied he broke his arm.

11. A chemist, a neuroscientist, and two biologists conducted several experiments.

The chemist and the neuroscientist came up with a new hypothesis, but the biologists offered an alternative account.

The chemist and the neuroscientist had recently won a Nobel prize for some important discoveries.

12. A drummer, a bassist, and two vocalists listened carefully while a song was playing.

The vocalists decided to change the lyrics after the drummer complained about the song's message.

The vocalists are no longer talking to the drummer, and the band might break up.

13. Two members of the marching band, a football player, and his girlfriend were going to dinner before prom.

The football player waited while his girlfriend and the others finished getting ready.

The football player's girlfriend got her dress at a thrift shop, so it cost very little.

14. Two actresses, a comedian, and a dog had a scene together in a play.

The comedian taught the dog tricks while the actresses watched.

The comedian has become very popular and all of his performances are sold out for the next two months.

15. Two supermodels, a businessman, and his wife drank red wine together on a yacht.

The businessman flirted with the supermodels while the wife rolled her eyes.

The businessman had dated five different models in the last month, and his wife was thinking of divorcing him.

16. Two parents, a daughter, and a son walked around together at the zoo.

The daughter stopped to buy some ice cream while the parents and the son looked at the lions.

The daughter loves to go to the zoo and has a season pass.

17. Two celebrities, a runway model and a rock star held a pre-party together before the big gala.

The celebrities talked to the rock star and then the rock star danced with the runway model.

The model just divorced her husband and is now dating the rock star.

18. Two CEOs, a strategist, and a trustee argued during a board meeting.

The trustee and the strategist agreed upon a solution but the CEOs preferred their own ideas.

The trustee had donated ten million dollars to the company, so he was unhappy when his opinion was not taken into consideration.

19. Two midfielders, a goalie, and a fullback made dessert together before the team dinner.

The goalie and the fullback baked a cake and then the midfielders decorated the cake with icing.

The whole team really cared about the soccer coach, and the cake was for his birthday.

20. Two chaperones, a freshman, and a senior class officer were having an argument at the school dance.

The chaperones were yelling at the senior class officer because she had been dancing inappropriately.

The chaperones yelled at most of the students at the dance and were known for being very strict.

21. Two bridesmaids, a groom, and a bride celebrated at the reception after a wedding.

The bride and groom danced while the bridesmaids looked on.

There was a rumor that the groom had cheated on the bride with one of the bridesmaids.

22. Two hall monitors, a janitor, and a police officer patrolled the hallways of the high school between classes.

The hall monitors asked the janitor to clean up after a food fight and then informed the police officer of the incident.

It took the janitor two hours to clean up the mess, and no one could identify the students involved in the food fight.

23. Two environmentalists, a zoologist and a philanthropist worked on a plan for saving the endangered African elephants.

After the environmentalists and the zoologist described the worsening situation, the philanthropist wrote a check for 23 million dollars.

The environmentalists and the zoologist have been working together for many years.

24. Two waitresses, a bouncer, and a DJ conversed during their break.

The bouncer playfully teased the waitresses, and then the DJ warned the bouncer about his inappropriate behavior.

The bouncer was known to be obnoxious, and the DJ didn't much care for him.

25. A city councilman, a treasurer, and an orator gathered together for a meeting.

The councilman and the treasurer wanted to improve their public speaking skills, so the orator was giving them advice.

The councilman's speeches improved significantly after meeting with the orator.

26. A queen, a princess and a prince prepared for the grand arrival of the king.

The queen applied make-up while the princess and the prince checked up on the festive meal.

The queen had spent the entire day primping for the arrival of the king.

27. A shark, a seal and an animal feeder were in the same tank together at the aquarium.

The shark attacked the seal while the animal feeder tried to distract it with shark food.

The shark bit the animal feeder on the leg, and he decided to sue the aquarium.

28. A fugitive, an undercover cop, and a schoolgirl were in the same train car.

The cop tackled the fugitive, and then the schoolgirl screamed.

The fugitive managed to escape, but the cop eventually caught up with him.

29. A farmer, a banker, and a mayor had a meeting on a farm.

The farmer talked to the banker while the mayor surveyed the farm.

The mayor wants to buy the farm and turn it into a community park, and she hopes to start construction this month.

30. A preacher, an assistant pastor, and an organist led praise worship during the service on Sunday.

The preacher said the prayer, and then the assistant pastor gave a reading.

The assistant pastor and the organist got married last month in the same church.

31. A golf pro, a novice player, and a caddy were on the golf course.

The golf pro demonstrated how to putt, and the novice imitated him.

The caddy thought that the golf pro was very patient, and the novice was really enjoying the lesson.

32. A university president, a campus police chief, and a dean spoke to the incoming class at orientation.

The chief's speech was sobering, but the other two speeches were more uplifting.

The university president is retiring this year, and the university has already found a replacement.

33. A rapper, his agent, and a dancer were going over the contract for a new music video.

The rapper talked to the agent while the dancer looked over the information about her salary.

The rapper had worked with the agent before, so he was confident that the contract was fair.

34. A physician assistant, a surgeon, and a patient discussed several treatment options in the emergency room.

The physician assistant and the surgeon recommended a surgery; however, the patient remained skeptical.

The physician assistant and the surgeon have been at the same hospital for nearly ten years and often work together.

35. A concierge, a pianist, and a flutist were engaged in an argument in the hotel lobby.

The pianist and the flutist stated they had made room reservations; however, the concierge claimed that they had not.

Later, the concierge was fired for being so rude because the pianist and the flutist complained to the manager about the horrible guest service.

36. A nanny, a dog walker, and a pickpocket were sitting in the same area at the park.

The dog walker punched the pickpocket after he stole the nanny's wallet.

The pickpocket got away, and the dog walker never found out whether he was ever arrested.
